# Patient adherence to medical treatment: a review of reviews

**DOI:** 10.1186/1472-6963-7-55

**Published:** 2007-04-17

**Authors:** Sandra van Dulmen, Emmy Sluijs, Liset van Dijk, Denise de Ridder, Rob Heerdink, Jozien Bensing

**Affiliations:** 1 NIVEL (Netherlands Institute for Health Services Research), PO box 1568, 3500 BN Utrecht, The Netherlands; 2 Utrecht University, Department of Psychology and Health, PO box 80140, 3508 TC Utrecht, The Netherlands; 3 Utrecht University, Department of Pharmacoepidemiology & Pharmacotherapy, PO box 80082, 3508 TB Utrecht, The Netherlands

## Abstract

**Background:**

Patients' non-adherence to medical treatment remains a persistent problem. Many interventions to improve patient adherence are unsuccessful and sound theoretical foundations are lacking. Innovations in theory and practice are badly needed. A new and promising way could be to review the existing reviews of adherence to interventions and identify the underlying theories for effective interventions. That is the aim of our study.

**Methods:**

The study is a review of 38 systematic reviews of the effectiveness of adherence interventions published between 1990 and 2005. Electronic literature searches were conducted in Medline, Psychinfo, Embase and the Cochrane Library. Explicit inclusion and exclusion criteria were applied. The scope of the study is patient adherence to medical treatment in the cure and care sector.

**Results:**

Significant differences in the effectiveness of adherence interventions were found in 23 of the 38 systematic reviews. Effective interventions were found in each of four theoretical approaches to adherence interventions: technical, behavioural, educational and multi-faceted or complex interventions. Technical solutions, such as a simplification of the regimen, were often found to be effective, although that does not count for every therapeutic regimen.

Overall, our results show that, firstly, there are effective adherence interventions without an explicit theoretical explanation of the operating mechanisms, for example technical solutions. Secondly, there are effective adherence interventions, which clearly stem from the behavioural theories, for example incentives and reminders. Thirdly, there are other theoretical models that seem plausible for explaining non-adherence, but not very effective in improving adherence behaviour. Fourthly, effective components within promising theories could not be identified because of the complexity of many adherence interventions and the lack of studies that explicitly compare theoretical components.

**Conclusion:**

There is a scarcity of comparative studies explicitly contrasting theoretical models or their components. The relative weight of these theories and the effective components in the interventions designed to improve adherence, need to be assessed in future studies.

## Background

The problem of non-adherence to medical treatment remains a challenge for the medical professions and social scientists. Their efforts to explain and improve patient adherence often appear to be ineffective. Although successful adherence interventions do exist [1-5], half of interventions seem to fail [6] and adherence theories lack sufficient explaining power. As a result of the widespread problem of adherence, substantial numbers of patients do not get the maximum benefit of medical treatment, resulting in poor health outcomes, lower quality of life and increased health care costs [7,8]. In spite of many advances made in adherence research, non-adherence rates have remained nearly unchanged in the last decades [9].

Overviews of the number of non-adherent patients can be found in various reviews [10-14]. DiMatteo found an average non-adherence rate of 24.8% of the patients [13] with the highest rate in patients with HIV, arthritis, gastrointestinal disorders and cancer. The lowest were in patients with pulmonary disease, diabetes and sleep disorders [13]. Measured with Electronic Measurement devices (EM), medication adherence appeared highest in cancer patients (80%), about 75% in many other diseases, for example in cardiovascular, infectious disease and diabetes, and lowest in chronic obstructive pulmonary diseases (COPD) (51%) and asthma (55%) [15]. Cramer found mean adherence rates of 58% and 65% among patients with psychiatric disorders and depression, respectively [11]. In general, adherence rates are higher among patients with acute conditions compared to patients with chronic diseases [16]. Consistent adherence among patients with chronic conditions is disappointingly low, dropping most dramatically after the first six months of therapy [17]. To tackle the problem of non-adherence, innumerable intervention studies have been performed in the last decades, many of which include interventions that are quite complex and time-consuming [18]. Unfortunately, even the most effective interventions appear to have only modest effects [19]. By now, the research seems to got stuck [20-23].

One reason for the slow progress in research and development into adherence is the lack of theories to predict and explain non-adherence adequately. It is as yet unclear whether some theoretical constructs might be more convincing than others in explaining and improving non-adherence [24-26]. We will try to deduce this knowledge from effective adherence interventions.

Apart from the search for effective interventions, this study explores which theories deserve to be developed further. So our main research questions are:

1. What are effective adherence interventions and how well do they improve non-adherence?

2 What are the theoretical perspectives that underlie effective adherence interventions and which of them are most promising for further research and development?

The first question will be answered by assessing the effectiveness of adherence interventions on the basis of systematic reviews. Regarding the second question, two ways exist to identify the theoretical perspectives underlying adherence interventions. Firstly, in a number of systematic reviews, the adherence interventions are categorized according to the underlying mainstream theories, for example, as either behavioural or educational or a combination of both [27-30]. Roter et al. clustered the interventions in four mainstream, global, theories: behavioural, educational, affective, or combinations of these [31]. By elaborating on their work, we try to analyse further the underlying theoretical principles. Secondly, some interventions are implicitly based on theoretical principles. For example when financial incentives are being used to improve adherence, the underlying theoretical perspective is behavioural because incentives are considered to act as positive reinforcers. Another class of intervention focuses on persuasive communication to improve adherence. As such, communication theories may underpin these interventions. Proceeding along this line of thought, the current study explores which theoretical perspectives, or combination of them, underlie effective adherence interventions. Because interventions to improve adherence are diverse in type and intensity and several reviews have already been written, we have chosen to summarize the results of existing reviews carried out so far. In that way, we hope to be able to capture a large number of primary intervention studies. We follow the procedure applied in former review of reviews [32,33].

## Methods

### Literature search

A systematic literature search was conducted in Medline, Psychinfo, Embase, the Cochrane Library of systematic reviews, and the NIVEL-catalogue, supplemented with manual searches of references. The main keywords were: patient compliance, patient adherence, treatment compliance and treatment drop-outs linked with the keywords, meta-analysis, systematic review and literature review. The searches focused on systematic reviews published between January 1990 and March 2005. Systematic reviews were defined as reproducible reviews, based on electronic literature searches and explicit criteria for the selection of the primary studies [34].

### Inclusion criteria

The searches yielded 918 references to adherence reviews (See Additional file 1 for exact results on the searches). The titles and abstracts of these reviews were screened. The full text articles in English were obtained and scored from the 214 reviews that seemed potentially suitable. Systematic reviews were included if the following selection criteria were met:

- The focus of the review is patient adherence to medical treatment – medication, diet, lifestyle changes or appointment keeping – for a diagnosed medical condition prescribed by a health care professional;

- The included reviews incorporate adherence interventions that use any of the broad range of adherence measurement tools ranging from direct observable behaviour, subjective self-reports, objective monitoring of medication usage, objective physiological/biomedical measures, health outcomes or combined adherence measurements;

- The effectiveness of adherence interventions is a main research question of the review;

- The reviewers conducted and reported electronic literature searches;

- The reviewers applied explicit criteria for the inclusion and exclusion of primary studies;

- The results of the review, that is the effects of adherence interventions, were reported in a quantifiable and tabulated way, for example with effect sizes and odds ratios.

All 214 reviews were scored by one reviewer (ES) and independently scored by one of two other reviewers (SvD, LvD) using the form presented as Additional file 2. The interrater agreement was 95%. The 5% in which there were disagreements, in total ten reviews, were resolved through discussion. A total of 38 systematic reviews met all the inclusion criteria and were included in the study.

### Exclusion criteria

Descriptive reviews were not included in the study. In addition, reviews on the following subjects were excluded:

- Primary prevention;

- Guideline adherence such as the adherence of health care professionals to protocols or guidelines;

- Reviews reporting only health outcomes without adherence measures.

### Data extraction

A data extraction form was used to assess the following characteristics of the reviews: the medical condition or disorder being studied; the type of adherence interventions; the period of literature searches; the number of primary studies; and the total number of patients involved in each review. In addition, we scored whether or not the reviewers had applied criteria concerning:

- randomisation procedures;

- measurements, electronic and otherwise, of adherence;

- minimum sample sizes in the primary studies;

- follow-up periods, in particular minimum ones;

- an analysis of the intention to treat, thereby including patients lost to follow-up;

- rating scales to assess the methodological quality of the primary studies;

- statistical pooling by meta-analytical computations.

A tabulated overview of the details per review is included as Additional file 3.

### Analysis

The reviews included were analysed in two steps. Firstly, effective adherence interventions mentioned in the reviews were identified on the basis of statistically significant differences. For this purpose three kinds of reviews were analysed: single-focus reviews; comparative reviews; and multiple-focus reviews. A single-focus review includes intervention studies that focus either on a cognitive, behavioural, or affective component, while a multiple-focus review includes interventions that incorporate cognitive, behavioural, and affective components. In some single-focus reviews the outcome of an intervention is compared to that of usual care, in others different interventions stemming from the same theoretical background are compared to one another. Reviews, without significant differences in effect between interventions, were analysed also. We were thereby able to discover whether or not a particular underlying theoretical perspective could explain the lack of significance – and would therefore have to be noted as being less promising. Secondly, we explored the theoretical perspective explicitly or implicitly underlying effective adherence interventions.

## Results

Table 1 gives a general overview of the 38 reviews included. The first 12 are single-focus reviews that include one type of adherence interventions, for example technical solutions such as, simplifying dosage or packaging, behavioural interventions, educational interventions, or social support. In the second 13 reviews, two or more types of interventions were analysed in comparison to one another. Most frequently a comparison was made between behavioural, educational, and complex or multi-faceted interventions. Each of the other 13 reviews cover multiple adherence interventions and were not restricted to one special type of intervention.

**Table 1:** The 38 included reviews and the focus on adherence interventions per review

	Disease/disorder	Technical	Behavioural	Educational	Affective	Other	Complex	Various
Single-focus reviews**)								

Buring SM et al., 1999 [36]*	Peptic ulcer	X						
Claxton AJ et al., 2001 [15]*	Various	X						
Connor J et al., 2004 [37]*	Various	X						
Iskedjian M et al., 2002 [38]*	Hypertension	X						
Richter A et al., 2003 [39]*	Various	X						
Yildiz A et al., 2004 [40]	Depression	X						
Giuffrida A et al., 1997 [42]*	Various		X					
Macharia WM et al., 1992 [43]*	Various		X					
Brown SA 1990 [45]*	Diabetes			X				
Devine EC 1996 [46]*	Asthma			X				
Devine EC et al., 1995 [47]*	Hypertension			X				
DiMatteo MR 2004 [52]*	Various					X ^1)^		

Comparative reviews**)								

Schedlbauer A et al., 2004 [58]	Hyperlipidemia	X		X		X ^2)^	X	
Schroeder K et al., 2004 [49]*	Hypertension	X		X		X ^3)^	X	
Merinder LB 2000 [54]	Schizophrenia		X	X				
Mullen PD et al., 1992 [51]*	Cardiac care		X	X				
Bender B et al., 2003 [27]	Asthma		X	X				
Peterson AM et al., 2003 [29]	Hyperlipidemia		X	X			X	
Peterson AM et al., 2003 [28]	Various		X	X			X	
Takiya LN et al., 2004 [30]	Hypertension		X	X			X	
Dolder ChR et al., 2003 [53]*	Schizophrenia		X	X	X		X	
Roter DL et al., 1998 [31]*	Various		X	X	X	X ^4)^	X	
Sharp J et al., 2005 [60]	Hemodialysis		X	X	X	X ^5)^	X	
Higgins N et al., 2004 [59]	Elderly			X			X	
Vergouwen ACM et al.2003 [50]*	Depression			X		X ^6)^		

Multiple-focus reviews**)								

Burke LE et al., 1997 [9]*	Cardiovascular							X
Dodds F et al., 2000 [35]*	Psychosis							X
Haynes RB et al., 2005 [6]*	Various							X
Morrison A et al., 2000 [41]*	Hypertension							X
Newell SA et al., 1999 [67]	Cardiovascular							X
Newell SA et al., 2000 [61]*	Cardiovascular							X
Nosé M et al., 2003 [55]	Schizophrenia							X
Pampallona S et al., 2002 [68]	Depression							X
Van Dam HA et al., 2003 [56]	Diabetes							X
Van der Wal MHL et al., 2005 [69]	Cardiovascular							X
Van Eijken M et al., 2003 [44]*	Elderly							X
Vermeire E et al., 2005 [57]	Diabetes							X
Zygmunt A et al., 2002 [48]*	Schizophrenia							X

Total	38	8	10	15	2	6	8	13

1) social support, 2) intensified care, 3) patient motivation, 4) provider directed interventions, 5) holistic approaches, 6) collaborative care

*) Reviews with significant differences between types of adherence interventions

**) Single-focus means that the interventions described in the review are all based on one model or theory; comparative means that two or more single-focus interventions are compared in the review; multiple-focus interventions are based on various models or theories

Many adherence interventions appear to be directed at the chronically ill. Twelve reviews concern cardiovascular problems or risks, three diabetes, two asthma/COPD, one haemodialysis and one peptic ulcer. Eight reviews address mental health problems, mainly schizophrenia and depression. Each of the remaining 11 reviews cover various diseases of which two reviews are restricted to the elderly population. Together, the 38 reviews cover 1,373 primary studies (range 4 – 153 studies per review) and 266,988 patients (range 543 – 57,528 patients per review). Twenty-eight of the 38 reviews included were published between 2000 and 2005. Sixteen reviews used meta-analytic computations (see Additional file 3 for further details). Two reviews included only studies in which adherence was followed up for at least six months [6,35]. In 23 of the 38 reviews, significant differences in the effectiveness of various adherence interventions were found. These were marked with an asterisk (*) in Table 1. Evidently, primary studies could have been included in more than one review. However, reviews may focus distinctly on one aspect of adherence behaviour, for example, appointment keeping, drop-out, or medication taking, or on one type of adherence intervention, for example medication packaging, financial incentives, or patient education. Having different points of view minimizes the doubling between the reviews.

### Reviews finding significant effects of interventions

#### Technical interventions

Technical adherence interventions, for example on dosage and packaging, are usually directed at simplifying the medication regimen. Most adherence interventions in this domain are aimed either at reducing the number of doses per day, for example through extended release formulations, or at reducing the number of different drugs in the regimen, for example by using fixed dose combination pills. Fixed dose combination pills are pills that include two or more drugs in fixed proportions in the same formulation, or blister packaging of several medications in a fixed combination, to be taken together.

The effects of technical adherence interventions have been assessed in several single-focus and comparative reviews [15,36-40]. Most reviewers arrive at the same conclusion that a less frequent dosage results in better adherence. These results are found across a variety of medical disorders and diseases such as peptic ulcer, hypertension, diabetes and cardiovascular disorders. Depression is an exception to this rule; the number of anti-depressant drugs does not seem to be related to the number of drop-outs [40].

Buring et al. performed a meta-analysis on adherence to antibiotic regimens for peptic ulcer disease caused by H. pylori [36]. The number of doses a day of such regimens may range from one to 16. Their analysis of 56 primary studies showed that adherence rates were higher with regimens containing three or fewer doses a day, compared to four to six doses a day (p = 0.001), seven to eleven (p = 0.009) or 12 or more (p < 0.0001). In this review the magnitude of effect was not mentioned.

The meta-analysis of Iskedjian et al. [38] also showed that the average adherence rate to antihypertension drugs was significantly higher for single daily dosage than for multiple daily dosage (91.4% versus 83.2%, p < 0.001). However, the longer the therapy lasted, the lower the adherence rates. For patients taking antihypertensive medication pill organizers and calendar packaging were also found to improve medication adherence [41]. Electronic vial caps improved adherence in a trial among elderly patients. These medication containers display the time when the container was last opened and beep when a dose is due to be taken. The odds ratios in the experimental group were about six times higher than those in the control groups [41].

The effectiveness of electronic devices on adherence was also investigated by Claxton et al. [15]. In their review they selected studies (N = 76) that used Electronic Monitoring (EM) devices to measure adherence. Adherence appeared to decline as the number of daily doses increased. Adherence to one dose was 79%, two doses 69%, three doses 65% and four doses 51%. Simplification of regimen by unit-of-use packaging also seems to improve adherence, but uncertainty remains about the size of these benefits [37]. All in all, there is consistent and robust evidence that simplifying medication dosage schedules leads to improved adherence [32] and, where feasible, reducing dose frequency may offer health outcome and cost benefits for the patients [39]. However, there are indications that the effects of this simplification become less the longer the treatment lasts.

#### Behavioural interventions

The most common behavioural interventions provide patients with memory aids and reminders, whether by mail, telephone, computer, or by home visits. Other classes of interventions consist of monitoring, by means of calendars or diaries, and providing feedback, support or rewards.

Giuffrida et al. reviewed 11 randomised trials, conducted in the United States, in which patients were paid for adherence in cash, gifts or vouchers. The incentives ranged from $5 to gifts worth nearly $1000. The results showed improved adherence in ten out of 11 studies (Odds ratios > 1.0). It remained unknown whether a cash payment or payment in kind was more effective. The authors argued that incentives can be cost-effective, if substantial benefits accrue, not only to the patient, but also to society at large. An example is to prevent the development of drug-resistant strains of infectious diseases or, in transplant patients, to prevent re-transplantation when patients adhere to their anti-rejection drugs [42].

Macharia et al. found that mailed reminders and telephone prompts were consistently useful for reducing the number of missed clinical appointments for the supervised administration of medical care [43]. The conclusions are based on their meta-analytic calculations of 23 randomised trials covering a fairly wide range of interventions and clinical settings. The most common intervention was simply a letter or telephone call a few days prior to the appointment to remind patients of the pending appointment. This proved to be effective in general medical populations (pooled Odds ratio 2.2). According to the authors, computerised reminders can be highly cost-effective. Van Eijken et al. found that a telephone-linked reminder system increased medication adherence among elderly people [44]. A review of 49 randomised trials in cardiac care found that enhancing self-efficacy, skill-training and self-monitoring are also successful strategies [9].

These reviews show that behavioural interventions not only have relevance for improving medication adherence, like most technical interventions have, but enhance adherence to other types of treatments as well.

#### Educational interventions

Education is a cognitive didactic approach that includes teaching and providing knowledge. There are different ways to educate patients: individual versus group education, face to face contact, audio-visually, in writing, by telephone, by e-mail or via home visits.

Three meta-analytic reviews focused on patient education in relation to chronic diseases. These included both types of diabetes, hypertension and asthma [45-47]. Together they cover 202 primary studies. The authors' main conclusions are that their analyses lend support to the effectiveness of patient education on knowledge, adherence and patient outcome. Knowledge showed the largest effect with a mean effect size of d_+ _1.05 in diabetes education [45] (the effect size 'd' represents the standardised mean difference between treatment and control groups, measured in standard deviation units; d_+ _is the average unbiased weighted effect size). The effects of knowledge, however, appear to diminish over time. Measured at two weeks after the intervention, hypertension education showed a large effect size on knowledge of d_+ _0.98, but declined to a medium effect size of d_+ _0.46 when measured at four weeks [47]. The reviews did not provide enough information about the educational programme to determine what types of programmes and educational strategies are most effective [45]. Zygmunt et al. found that educating patients in concrete problem solving and motivational techniques increased medication adherence among schizophrenic patients [48]. In their review of 39 studies, the authors also found that psycho-educational programmes, which are common in clinical practice, were typically ineffective [48]. Education did appear to increase patient adherence in asthma (effect size d_+ _0.70) and hypertension (effect size d_+ _0.49). In diabetes, adherence to dietary regimens also improved with education (effect size d_+ _0.57), but the effects on weight loss were much smaller (effect size d_+ _0.17) [45].

Other reviewers found that education had positive effects on metabolic control [45], blood pressure [47] and asthma [46]. According to Devine the positive effect of education is probably attributable to the fact that many of the educational programmes included instructions on appropriate medication usage as well as self-care activities [46]. However, Schroeder et al. compared four types of adherence interventions in hypertension patients from 38 trials and found that the most effective intervention was not education but dosage simplification. Reducing the number of daily doses of blood pressure lowering medication increased adherence by eight to twenty percent [49].

An effective adherence intervention in primary care turned out to be collaborative care [50]. Collaborative care was defined as a systematic approach that improves patient education through mental health professionals or other care providers, such as nurses in primary care, playing an active role [50]. Collaborative care was tested against patient education in a review of 19 randomised trials, of which 13 were in primary care. Nine of the 13 primary care studies showed significant differences in adherence between intervention and usual care groups, with an increased adherence of approximately 25%. Better depression outcomes were achieved as well, especially in patients suffering from major depression, who were prescribed adequate dosages of antidepressant medication [50].

Mullen's meta-analysis included 28 controlled trials on cardiac patient education programmes [51]. Patient education was broadly defined and encompassed didactic, as well as, behavioural approaches. Many cardiac programmes were intensive and consisted of large numbers of contacts, for example in supervised cardiac exercise programmes. The effects were seen in clinical and behavioural outcomes. The average sizes of the effect were 0.51 for blood pressure, 0.24 for mortality, 0.19 for diet and 0.18 for exercise. Smoking cessation and drug adherence did not change significantly. The trend was for behaviourally-oriented interventions to have larger effects [51]. However, the difference with didactic interventions did not reach statistical significance, because, according to Mullen, intensive affective interventions were applied in the didactic programmes.

Unfortunately, no comparison of two or more types of interventions was carried out within the studies in order to test the effectiveness of different types or components of interventions [46]. Besides, subgroup analyses or pooling of the results were not always allowed due to the heterogeneity of the samples as defined by Hedges' test of homogeneity. A major weakness of the existing research is under-reporting of key aspects of the studies, for example, the duration of the treatment [47].

#### Social support interventions

A meta-analysis of 122 studies, conducted by DiMatteo, aimed at assessing which type of social support, either practical, emotional or undifferentiated, has the strongest relationship with adherence [52]. It appeared that practical social support yielded significantly higher effects than emotional and undifferentiated support. The standardized odds Ratio was 3.60 (2.55–519). There appeared to be a 0.65 SD difference in adherence between patients receiving practical support for their treatment regimen and those not receiving such support. Unfortunately, it is not yet understood how social support contributes to health and which factors moderate and mediate this relationship.

#### Structural interventions

An example of a structural or organizational intervention is a programme of care at the place of work to manage hypertension, administered by specially trained nurses as described by Morrison et al. [41]. They found a small but significant improvement in adherence and blood pressure. Additional strategies, such as a disease management programme aimed exclusively at the non-adherent patients, yielded no significant improvements [41]. Another example of structural interventions is provided in the review by Zygmunt et al. on community-based rehabilitative intervention programmes for schizophrenic patients [48]. The authors found that such interventions, targeted specifically to non-adherence problems, were twice as effective as more broadly based interventions.

#### Complex or multi-faceted interventions

Among the category of complex interventions, the findings of Haynes et al. deserve special attention [6]. They updated their review of 2002 and added 25 recent studies. They came to three conclusions on the basis of 57 un-confounded randomised trials that reported adherence and treatment outcomes with a follow-up period of at least six months. Firstly, less than half (45%) of the interventions resulted in improved adherence and only 33% in better treatment outcomes. Secondly, those interventions that were effective for long-term care were exceedingly complex and labour-intensive. Thirdly, even the most effective interventions did not lead to large improvements in adherence and treatment outcomes [6].

Roter et al. conducted meta-analytic computations in their review (153 studies) [31]. They found that no single strategy or programmatic focus showed any clear advantage over the other. Comprehensive interventions, combining cognitive, behavioural and affective components, were more effective than single-focus ones (ES 0.34). Affective components concern the provider-patient relationship and refer to issues such as empathy, attentiveness, care, concern or support. The same results were reported by Dolder et al. in a review on schizophrenia [53]. Among schizophrenic patients, interventions of a purely educational nature were the least successful at improving adherence to anti-psychotic medication [53], and behavioural components seem to be needed [54]. The intensity and duration of the interventions did matter, according to Dolder et al. Interventions reporting an improvement in adherence had a median of eight sessions, while those interventions without gains in adherence had a median of three sessions [53]. Written materials were weaker (ES 0.12) than other educational interventions in Roter's review, but written, mailed, reminders (ES 0.21) were as effective as telephone reminders (ES 0.19) in keeping appointments.

Roter et al. concluded that behavioural and educational approaches were equally effective but they also suggested that the addition of affective components enhances the effectiveness of the interventions [31]. The variability in study design, along with the multitude of adherence definitions and assessments, precluded reviewers from performing a meaningful meta-analysis [53]. Besides, the differences in adherence measures and definitions of adherence, create complications when trying to compare changes in adherence among studies and when calculating mean, non-adherence, rates.

### Reviews finding no significant effects of interventions

In fifteen reviews no differences in effectiveness were found. Although some effective interventions were found in most reviews, the reviewers did not find statistical differences between the interventions, or else the authors were reluctant to recommend one intervention over others, due to a lack of evidence. We give here some examples.

#### Overall small effects

No single intervention emerged as a predictor of the overall effect of treatment in four meta-regression analyses [23,28,30,55]. For example, in a thorough meta-analysis (61 studies) Peterson et al. found no significant variation between the different intervention categories. Educational interventions showed an effect size of 0.11, behavioural interventions 0.07, and the combined interventions, 0.08 [28]. Takiya et al. also only found a small and insignificant degree of effect of 0.04 for behavioural interventions in their meta-analytic review of antihypertensives (16 studies). Van Dam et al. concluded that patient-focused interventions among people with type 2 diabetes were more effective than provider-focused ones, but the various patient-focused interventions hardly differed in their effectiveness [56]. The meta-analysis of Vermeire et al. (21 trials) showed small effects on a variety of outcomes but no highlights appeared [57]. The authors' conclusion is that: "The current efforts to improve or to facilitate adherence of people with type 2 diabetes to treatment recommendations do not show significant effects nor harm. The question whether any intervention enhances adherence to treatment recommendations in type 2 diabetes effectively, thus still remains unanswered" [57]. In four other reviews including two reviews on hyperlipidaemia [29,58], a review on asthma [27] and a review on medication adherence among the elderly [59], no intervention could be recommended due to the poor methodological quality of the studies reviewed.

#### Overlapping components of intervention

The systematic review of Sharp et al. (16 studies) aimed at assessing effective components of psychological interventions for improving the adherence of patients receiving haemodialysis [60]. The components of such interventions were intended, for example, to modify health beliefs, apply stages of change theory, self-efficacy training or self-monitoring. The results showed that such psychological interventions indicate some success [60]. Superior theories were not found. However, due to the considerable number of components included in any one study, and the overlap between the components used in different types of interventions, it was not possible to examine the efficacy of different intervention components. Therefore it is difficult, according to the authors, to establish the components of treatment responsible for clinical change [60].

### Comparison of reviews with and without significant effects of interventions

Finally, no obvious differences were seen between the 15 reviews without significant differences, and the 23 reviews with significant differences between interventions. These two sets of reviews did not differ in respect to the methods applied. In both sets almost half of the reviewers selected only randomised trials (6/15 and 11/23, respectively) and in both sets about 40% of the reviews used meta-analytical computations (6/15 and 10/23, respectively). In addition, no differences were found in the diseases or disorders between the two sets of reviews. There were two differences that did emerge. Firstly, only one review in the set of 15 reviews addressed technical solutions, compared to five of the 23 reviews. Secondly, the set of 15 reviews was of a more recent date; fourteen of the 15 reviews (93%) were published between 2000 and 2005 against 61% in the other set (14/23). So far the evidence-base for these type of interventions seems to be the strongest. This is demonstrated by the low number of reviews that found no significant effects of technical interventions. The relatively large number of reviews that did point to the effectiveness of technical interventions (see also Table 2) also pointed to this conclusion.

**Table 2:** Reviewers who found effective adherence interventions

Technical interventions	Behavioural interventions	Educational interventions	Other interventions	Multifaceted/Complex
Buring, 1999 [36]	Burke, 1997 [9]	Brown, 1990 [45]	DiMatteo,2004 [52]	Dolder, 2003 [53]
Claxton, 2001 [15]	Dodds, 2000 [35]	Devine, 1995 [47]	Newell,2000 [61]	Haynes,2005 [6]
Connor, 2004 [37]	Giufffrida,1997 [42]	Devine, 1996 [46]		Roter, 1998 [31]
Iskedjian, 2002 [38]	Macharia, 1992 [43]	Mullen,1992 [51]**		Vergouwen,2003 [50]*
Morrison, 2000 [41]	VanEijken,2003 [44]	Zygmunt,2002 [48]		
Richter, 2003 [39]				
Schroeder,2004 [49]				

Total 7	5	5	2	4

*) We consider collaborative care to be a multifaceted intervention

**) Intensive cardiac patient education can also be considered multifaceted or complex

### Underlying theoretical perspectives of the most effective intervention methodologies

We found 23 reviews with significant differences between interventions. An overview of the main findings of these reviews are summarized in Table 2. Evidently, effective adherence interventions were found in four types of adherence interventions: technical, behavioural, educational and multi-faceted or complex interventions. The fifth type, consisting of affective interventions, has not been investigated in isolation. Table 2 shows that technical solutions, mainly consisting of simplifying the dosage and packaging, were effective in seven reviews. Behavioural approaches were effective in five reviews, educational approaches also in five reviews and complex or multi-faceted interventions in four reviews. Two reviews found some evidence for social support [52] and partner-focused strategies [61].

We will now look in more detail on the theoretical principles underlying the adherence interventions for which we found most evidence: technical, behavioural and educational interventions. A general observation is that most interventions are eclectic in nature and not strictly representative of one theoretical model. However, some uniformity can be discovered and theoretical constructs can, sometimes, be clearly identified.

### Theoretical perspectives underlying technical solutions

Technical adherence interventions imply a simplification of the regimen. There is robust evidence that such simplifications, regarding, for example, dosage and packaging, improve patient adherence. These technical solutions reflect the bio-medical model or perspective in which medical experts seek solutions for patients' problems [62]. Initially, the bio-medical model sought the reason for non-adherence in, deviant, dispositional characteristics of the patient, for example, in personality characteristics or cognitive impairments. However such factors were hardly found [63]. The factors that were found concerned the severity of symptoms and features of treatment or side effects. These findings have motivated the development of technological 'fixes' to enhance compliance [62].

The fact that simplification of regimen improves patient adherence appeals to one's intuition. It seems a practical and logical solution. Theoretically, however, the operating mechanism in this bio-medical perspective is all but clear. What exactly causes the patient to change his or her behaviour? Is taking one pill so much easier than taking two? According to Claxton et al., the findings reinforce the principle of simplicity [15]. However, they gave no further theoretical explanations. Perhaps the lack of a sound explanation is one of the reasons why some reviewers sometimes categorize technical adherence interventions under behavioural approaches [31]. Although the quest for technical solutions is as old as mankind itself, up till now sound theoretical explanations for the effectiveness of simplification are lacking. Finding such explanations seems a first challenge for any development of theory. Perhaps medical and social psychology scientists should connect with scientists from other fields, for example human engineering, ergonomics, and technical sciences, in order to collaborate in the interests of exploring the theory further.

### Theoretical perspectives underlying behavioural interventions

According to our findings, interventions based on reminders and incentives can be successful in improving patient adherence. These interventions represent the powerful principles of behavioural theories. From the perspective of these theories, human behaviour depends on stimuli or cues that elicit certain responses and on the rewards that reinforce behaviour. Reminders can act as cues or stimuli, and incentives as rewards, being all kinds of positive consequences of the behaviour. These are the main, and best known, first principles of behaviour theory. The behaviour may be learned by gradual shaping or forming a pattern of behaviour. Maintenance of the desired behaviour may occur by automation after sufficient repetition [62].

Our findings show that reminders are successful in improving appointment keeping. As such, sending reminders may be considered to be one of the most inexpensive adherence interventions. Reminders are becoming even more easy to apply with the help of information technology. It should be noted, however, that patients' actual behaviour in taking medication seems less amenable to reminders. This remains a question for future research. Our findings only concern the original basic principles of behaviour theory, stimuli and rewards. Over time, however, the behavioural approaches have been widened. Bandura incorporated principles from social learning theories, for example modeling and vicarious learning, that is learning by watching, listening or reading. He also added the concept of self-efficacy, the confidence in one's capacity to perform the desired behaviour [64]. Adding these concepts is assumed to make the behavioural approaches more powerful. However, in our sets of reviews these concepts were not examined in isolation so the effectiveness of the various components could not be assessed.

### Theoretical perspectives underlying educational interventions

Education originally refers to a cognitive didactic approach, but nowadays appears to be an overall concept. Educational interventions are defined as any intervention given with the intent of improving the person's ability to manage his or her disease [45]. Behavioural principles, such as reinforcement and feedback, are increasingly incorporated into such educational interventions. In order for education interventions to be effective they should be tailored to the patient's needs and situation. In addition, attention should be paid to the quality of the relationship between the provider and the patient [65]. This makes the concept of patient education a complex one, and one that does not solely refer to a cognitive or didactic theoretical model.

Clearly, patient education may contain components of more than one theoretical approach. Unfortunately, we do not know exactly which components contribute to the success of the educational interventions because the reviews we examined did not make clear which elements were present. Sometimes the content of educational interventions was not described or the descriptions were too broad to deduce the components. For example, when the interventions were introduced as making use of patient counseling and self-management programmes. Educational interventions seem more often denominated by their form and their purposes or goals than by their content.

Leventhal et al. [62] distinguished three theoretical approaches underlying different forms of educational interventions: a) communication perspectives, b) cognitive perspectives, and c) self-regulation perspectives. In educational interventions that focus on the transfer of information and knowledge about the disease and its management, the theoretical perspective can be found in communication models. These models emphasize conveying the message by trusted and affective messengers. The patient should be informed adequately. Adequate not only implies that patients understand and retain the message, additional conditions are required for the communication to be effective in changing patients' attitudes and motivations to adhere to the treatment regimen. Patients should believe in the message as well as in the messenger. They should accept the information on the treatment regimen and the benefits of adherence behaviour. The emphasis is on information about 'why' adherence is needed to influence patients' attitudes and motivations. Other factors, external to the message itself, enhance acceptance of the message, such as the alliance with the therapist [53], and affective components including, for example, the practitioner's empathy, friendliness, interest and concern. Additional information can also facilitate behavioural change, for example information about ways to incorporate the behaviour into the patient's daily routines. In educational interventions that concentrate on changing patients' (dysfunctional) ideas and perceptions, cognitive models form the underlying theoretical perspective. The cognitive models emphasize patients' perceptions and beliefs as motivating factors for behaviour. Cognitive models focus on a cost/benefit analysis as a motivating factor for taking action. These models assume that health-related behaviour is determined by perceived health threats and the benefits of health behaviour. The basic dimensions of such a health belief model are the perceived probability and severity of the threat, on the one hand, and the perceived benefits of health behaviour and the barriers to such behaviour, on the other. Weighing the benefits and barriers and the consequences of various behaviours provides the motivation for the actions to be taken. Such weighing is not based on objective rational computations, but on the individual's own subjective perceptions of the pros and cons. Motivation is also determined by perceived social, group, norms and the perceived social consequences regarding the behaviour and its acceptability. In educational interventions that aim at self-management, the underlying perspective are self-regulation models. These models emphasize the patients themselves as active problem solvers [66,67]. Patients try to close the gap between the current health status and a goal. In self-regulative models behaviour is considerably influenced by patients' subjective experiences and emotions. Behaviour depends on several factors. These include: the patient's perceptions of the current status and the goal; the patient's plans for changing the current status to reach the goal, or coping; and the patient's appraisal of the progress in reaching the goal. If goals are not reached, patients may change their perceptions, or the labeling of the status, or their way of coping. Patients' ways of coping depend on cognitive considerations, for example, the perceived identity of health threats and their labeling of the symptoms and potential causes. Parallel to these cognitive processes, emotional reactions may exist and interact. Patients will also label these emotions and their causes, as well as their coping aims, to control or diminish, often stressful, emotions. Both cognitive and emotional ways of coping may be triggered by internal stimuli, for example, symptoms, or external ones, such as media messages [62].

In summary, components of these three theoretical approaches are part of an educational approach to 'improve the person's ability to manage his or her disease' [45]. Education often appears to reflect an eclectic approach. From the results of our study it is as yet unclear whether these three theoretical approaches are equally powerful, or powerless, in improving adherence. Intuitively, each seems to be plausible for explaining adherence behaviour. However, the relative weight of these theories, or the effective components in educational interventions designed to improve adherence, could not be assessed.

## Discussion

The aim of this study was to explore which types of adherence interventions, and their underlying theoretical perspectives, are promising for future research and development. Our motive for this study was the slow progress adherence research has made over thirty years and the disappointing effects of many adherence interventions. Although our study does not allow for firm conclusions, the findings may inspire new directions or ideas. The review studies selected were of a high quality, yet more well-designed studies are needed to formulate robust recommendations [56,68,69]. Comparisons are difficult, due to differences in adherence measures, in interventions and in study populations [35]. Besides, since we found only two reviews with a follow-up of six months or more [6,35], it is not possible to indicate what kinds of interventions are capable of fostering long-term improvements in adherence. More long-term evaluations are therefore recommended to establish which interventions maintain their effect over time [55].

The 'review of reviews' methodology we employed in the present study proved to be a valuable tool for gathering relevant studies. However, this methodology should be used carefully. Even though most of the reviews that we found incorporated different outcome measures, patient populations and time periods, doubling of primary studies between reviews can not be ruled out. Depending on the amount of doubling, this could bear consequences for the validity of our study. In a post hoc analysis we therefore examined the potential effect of doubling by comparing the lists with references to primary studies underlying reviews that were as much as possible comparable in view of topic, population and time period and looked at the number of doublings between these reviews. The analyis showed that doubling was neglectable. For example, only three of the eight primary studies reviewed by Iskedjian et al. on increasing adherence to hypertensives [38] were also included in the review performed by Schroeder et al. [49] and Morrison et al. [41]. Takiya et al.'s review even included only two of these eight primary studies [30]. Likewise, none of the nineteen primary intervention studies on adherence in antidepressants reviewed by Vergouwen et al. [50] were included in the review by Yildiz et al. [40] and only one was also included by Pampallona et al. [68]. These examples indicate that there is some overlap, but we considered it too small for questioning the validity of our findings. Another limitation of a 'review of reviews' was described in the special report [32]. It concerns the fact that the primary studies were selected for inclusion by the authors for their own systematic review according to criteria that may not match the intended characteristics for our review. The more strict the criteria the less likely a study will be included in the final body of evidence [32].

## Conclusion

The overall results of our study indicate some obvious findings concerning the current adherence interventions and the underlying theoretical perspectives:

Firstly, there are effective adherence interventions based on technical solutions such as simplifications of dosage and packaging, which lack an explicit theoretical explanation of its operating mechanism. This seems a first challenge for any development of adherence theory. Perhaps medical and social psychology scientists should connect with scientists from other fields, for example human engineering, ergonomics, and technical sciences, in order to collaborate in the interests of exploring the theory further;

Secondly, there are effective adherence interventions, such as incentives and reminders, which clearly stem from behavioural theories. Behavioural interventions seem specifically worthwhile for the subgroup of non-adherent patients who regularly forget to take their medication. It should be borne in mind, however, that patients' actual behaviour in taking medication seems less amenable to change by using reminders;

Thirdly, there is a scarcity of comparative studies explicitly contrasting theoretical models or their components. From the results of our study it is as yet unclear whether the biomedical, behavioural or educational models are more or less powerful in improving adherence. The relative weight of these theories and the effective components in the interventions designed to improve adherence, need to be assessed in future studies.

## Competing interests

The author(s) declare that they have no competing interests.

## Authors' contributions

JB conceived the study and developed its design. SvD co-ordinated the research team and drafted the manuscript together with ES. The other authors, LvD, DdR and RH, contributed substantially to the analysis and interpretation of the data and commented on the drafts of the manuscript.

## Pre-publication history

The pre-publication history for this paper can be accessed here:



## Supplementary Material

Additional File 1 **Search strategies and results**. Search strategies for each database. Click here for file

Additional File 2 **Checklist for reviews**. Checklist for inclusion and exclusion of reviews. Click here for file

Additional File 3 **Review of reviews adh table**. This file gives detailed tabulated information on the included reviews. Click here for file
