# Supplementary material for: Patient adherence to medical treatment: a review of reviews
Source: BMC Health Serv Res. 2007 Apr 17;7:55. doi: 10.1186/1472-6963-7-55 (PMC1955829; doi:10.1186/1472-6963-7-55)
Supplement: Additional File 1 — Search strategies and results. Search strategies for each database. [file 1472-6963-7-55-S1.doc]

Additional file 1 - Search strategies

Search strategy COCHRANE DATABASE dd. 1-2-05

#1 854 compliance:ti (1990 to current date)

#2 396 adherence:ti (1990 to current date)

#3 1255 #1 OR #2

#4 10266 screening:ti OR (guideline:ti NEXT adherence:ti) OR (reproductive:ti NEXT control:ti) OR prevention:ti (1990 to current date)

#5 1178 #3 NOT #4

Excluded: 1067 in Cochrane Central Register of Controlled Trials

I ncluded:

5 titles in Cochrane Database of Systematic Reviews – Complete Reviews

16 titles in Database of Abstracts of Reviews of effects – Abstracts of quality assessed systematic reviews

3 titles in Database of Abstracts of Reviews of effects – Other reviews

A total of 24 review titles included in the original literature list.

# Search strategy EMBASE dd. 02-03-05

No. Records Request

1 26430 "patient-compliance"/ all subheadings

2 85283 explode "practice-guideline"/ all subheadings

3 37089 explode "mass-screening"/ all subheadings

4 208039 explode "agents-acting-on-the-genital-system"/ all subheadings

5 255841 explode "prevention"/ all subheadings

6 546750 #2 or #3 or #4 or #5

7 20286 #1 not #6 = Compliance

21 569340 “review”/ all subheadings

22 3060 “systematic-review”/ all subheadings

23 20324 “meta-analysis”/ all subheadings

24 582585 #21 or #22 or #23

25 550716 review in dt

26 0 meta analysis in dt

27 0 review in pt

28 0 meta analysis in pt

29 582642 #24 or #25 : = Reviews.)

30 3357 #7 and #29 (Combination of Compliance & Reviews)

**Refinements 1**

31 194697 “drug-efficacy”/ all subheadings

32 50069 explode “drug-metabolism”/ all subheadings

33 30531 “drug-potency”/ all subheadings

34 20655 “drug-potentiation”/ all subheadings

35 4211 “drug-intoxication”/ all subheadings

36 280888 #31 or #32 or #33 or #34 or #35 (Excluded aspects)

37 2485 #30 not #36 (Combination Compliantie & Reviews & excluded aspects).

**Refinements 2**

38 22294 #22 or #23 (Restricted reviews: ‘systematic review’ en ‘meta-analysis’)

39 345 #7 and #38 (Compliance and (restricted) reviews).

40 205 #39 not #36 (Idem, and excluded aspects)

41 691492 patient

**Refinements 3**

42 50371 compliance

43 3867 patient compliance in dem (Compliance & ‘patient compliance’ as major keyword)

44 452 #29 and #43 (Compliantie & Reviews).

**A total of 452 titles included in the original literaturelist**

Search strategy PSYCHINFO dd. 1-2-05

**Search History**

#4 5009 ("Compliance-" in MJ,MN) or ("Treatment-Compliance" in MJ,MN)

#5 880 "Treatment-Dropouts" in MJ,MN

#6 5751 ("Treatment-Dropouts" in MJ,MN) or (("Compliance-" in MJ,MN) or ("Treatment-Compliance" in MJ,MN))

#8 11202 ("Literature-Review" in MJ,MN) or ("Meta-Analysis" in MJ,MN)

#9 5323 REVIEW in DT

#10 16520 (REVIEW in DT) or (("Literature-Review" in MJ,MN) or ("Meta-Analysis" in MJ,MN))

#11 47 ((REVIEW in DT) or (("Literature-Review" in MJ,MN) or ("Meta-Analysis" in MJ,MN))) and (("Treatment-Dropouts" in MJ,MN) or (("Compliance-" in MJ,MN) or ("Treatment-Compliance" in MJ,MN)))

**A total of 47 reviews included in the original literature list.**

Search strategy PUBMED dd. 1-2-05

#2
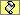
 17638 "Patient Compliance"[MeSH] Field: All Fields, Limits: Publication Date from 1990/01/01

#3 103936
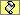
"Guideline Adherence"[MeSH] OR "Mass Screening"[MeSH] OR "Reproductive Control Agents"[MeSH] OR "Primary Prevention"[MeSH] Limits: Publication Date from 1990/01/01

#4 16323
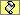
 #2 NOT #3 Limits: Publication Date from 1990/01/01

( = Compliance)

#9 7301
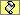
 "Review Literature"[MeSH] OR "Meta-Analysis"[MeSH] Limits: Publication Date from 1990/01/01

#10 9867
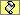
review literature[pt] OR meta-analysis[pt] Limits: Publication Date from 1990/01/01(= Meta-analysis[pt] )

#15
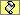
9867 meta-analysis[pt] Limits: Publication Date from 1990/01/01

( = meta-analysis[pt] )

#16 814527
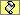
review[pt] Limits: Publication Date from 1990/01/01

#17
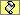
95046 literature AND (review OR reviews) Limits: Publication Date from 1990/01/01

#18
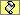
 70057 #16 AND #17 Limits: Publication Date from 1990/01/01

#19
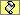
85548 #9 OR #15 OR #18 Limits: Publication Date from 1990/01/01( = Reviews).

#20
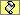
 405 #4 AND #19 Limits: Publication Date from 1990/01/01

A total of 405 reviews included in the original literature list.

Results of literature searches: 928 reviews.

After removing duplicates a total of 918 reviews in the original literature list.
