# Supplementary material for: Patient adherence to medical treatment: a review of reviews
Source: BMC Health Serv Res. 2007 Apr 17;7:55. doi: 10.1186/1472-6963-7-55 (PMC1955829; doi:10.1186/1472-6963-7-55)
Supplement: Additional File 2 — Checklist for reviews. Checklist for inclusion and exclusion of reviews. [file 1472-6963-7-55-S2.doc]

# Additional file 2 - Checklist for inclusion and exclusion of reviews

Author and year of publication ………………………………………………….

| Subject of the review  Adherence to medical treatment prescribed by health professional | yes | no | unclear |
| --- | --- | --- | --- |
| Research question  Effectiveness of interventions/measures to increase adherence |  |  |  |
| Literature search  Electronic literature searches |  |  |  |
| Primary studies  In- and exclusion criteria are applied to primary studies |  |  |  |
| Review method  Meta analysis |  |  |  |
| Results of review  Reported in quantitative and tabulated way |  |  |  |

## Final judgement

0 Inclusion 0 Exclusion 0 Unclear

## Main reason for exclusion

### Focus of the review on Methods of review

0 prevention 0 descriptive review

0 (new) medication/treatment effects 0 qualitative results

0 guideline adherence 0 literature searches unclear

0 on outcome (without adherence) 0 inclusion criteria studies unclear

0 on factors related to adherence 0 other………………………………

0 on magnitude of adherence

0 other:………………………………………
