# Supplementary material for: Patient adherence to medical treatment: a review of reviews
Source: BMC Health Serv Res. 2007 Apr 17;7:55. doi: 10.1186/1472-6963-7-55 (PMC1955829; doi:10.1186/1472-6963-7-55)
Supplement: Additional File 3 — Review of reviews adh table. This file gives detailed tabulated information on the included reviews. [file 1472-6963-7-55-S3.pdf]

Tabulated overview of included reviews

First author	Number of RCT's	Review / Meta	Adherence Interventions	Results / Improvement	Authors' conclusions	Authors' recommendations for practice	Authors' recommendations for research	Authors' other remarks
Bender B. et.al., 2003<br>Asthma<br>Search 1992-2003<br>16 studies<br>3606 patients		Review	1) Educational<br>2) Behavioral	1) Improvements in 9/16 studies<br>2) Self reported adherence was often not accompanied by treatment success	1) There is little concurrence in reports of adherence interventions<br>2) Many interventions involved a significant amount of professional time yet few were very effective	Research into innovative interventions, that are brief easily implemented and can be tailored to individual patients and diverse clinical settings	Inclusion of hard-to-reach and of poor patients, use valid measures of adherence at intervals, sufficient to establish enduring benefit	The need for methodological sound studies of innovative approaches to adherence promotion is evident
Brown SA, 1990<br>Diabetes (I and II)<br>Search 1961-1989<br>82 studies<br>5348 patients		Meta	Various interventions	1) Knowledge effects: ES range 0.49-1.05<br>2) Self care effects: ES range 0.17-0.57<br>3) Metabolic control: ES range 0.16-0.41<br>4) Psychological outcomes ES 0.27	1) This meta-analysis lends support to the effectiveness of diabetes patient education in improving patient outcomes<br>2) It is unknown what types of educational strategies are most effective	Ensure that more diabetes patients have access to patient education programs	1) Description of study sample and sampling procedure<br>2) Description of interventions	Details of most studies related to how the research was conducted and little attention was given to describing the interventions
Buring SM et.al., 1999<br>Peptic ulcer (H.pylori)<br>Search 1990-1996<br>63 studies<br>5996 patients	63 RCT	Meta	The number of daily doses	1) The more dd's, the more dropouts<br>2) ADR-documentation, more dropouts<br>3) Symptomatic relief, less dropouts	High number of doses per day results in higher discontinuation	An ideal regimen would have one or two drugs and fewer than three doses/day	Future trials should include:<br>minimum compliance standards;<br>adverse effect diaries or interviews;<br>intent-to-treat analyses	None
Burke LE et.al., 1997<br>Cardiovascular (+ risks)<br>Search 1977-1997<br>46 studies<br>29.485 patients	46 RCT	Review	Various interventions	13 strategies were successful, among them:<br>behavioral skill training<br>self-monitoring<br>telephone/mail contact<br>self-efficacy enhancement external cognitive aids etc.	1) This review reflects the progress made over two decades in compliance measurement and research<br>2) The majority of the interventions have not been tested in controlled studies for comparative efficacy	1) One of the challenges is to reduce the gap between compliance in clinical trials (not ideal) and compliance in clinical practice (even lower)<br>2) Focus interventions on the subgroups identified as non-adherent	1) Test comparative efficacy of successful strategies<br>2) Focus on subgroups of non-adherent patients	Despite the progress made in compliance research, compliance rates have remained nearly unchanged during the last 20 years

Tabulated overview of included reviews

First author	Number of RCT's	Review / Meta	Adherence Interventions	Results / Improvement	Authors' conclusions	Authors' recommendations for practice	Authors' recommendations for research	Authors' other remarks
Claxton AJ et.al., 2001<br>Various diseases<br>Search 1986-2000<br>76 studies	76 RCT<br>MEMS (elec.monit.)	Review	Number and timing of doses	1 dose, compliance = 79%<br>2 doses, compliance = 69%<br>3 doses, compliance = 65%<br>4 doses, compliance = 51%	1) The number of doses per day is inversely related to compliance<br>2) The mean dose-taking compliance rate ranged from 70% to 80% in all but respiratory disease, indicating the similarity of compliance rates across therapeutic areas	1) Simpler, less frequent dd's, better compliance<br>2) EM-devices could be used in clinical practice to evaluate the reason for lack of expected treatment effect	1) Further recognition of the influence of medication compliance on health outcomes will enhance research in this area<br>2) Further research is needed about dose timing	Monitoring compliance with EM-device has not been demonstrated to influence compliance; such monitoring is not sufficient to change behavior
Connor J. et. al., 2004<br>Various diseases<br>Search 1966-2003<br>15 studies<br>3.561 patients	15 RCT	Review	1) Packaging (unit-of-use packaging)<br>2) Fxed-dose combination pills	1) Improvements in 3/15 studies<br>2) Improved adherence in 7/13 studies	1) Fixed-dose combination pills and units-of-use packaging are likely to improve adherence<br>2) Uncertainty remains about the size of these benefits	1) The paucity of reliable evidence about effective strategies for improving adherence is extraordinary given the investment in assessing the efficacy of separate medications and the number of individuals taking multiple medications<br>2) Development and evaluation of fixed-dose combination products for developing countries	Improve methodological quality: larger samples;<br>follow-up at least 6 months;<br>blinding subjects and assessors;<br>dealing with subject losses	There seems to be little incentive for companies to invest in combinations of off-patent products that might compete with on-patient monotherapy
Devine EC, 1996<br>Asthma<br>Search 1972-1993<br>31 studies<br>1.860 patients	18 RCT	Meta	Psycho-educational care	1) Improved adherence ES is =.78<br>2) Eight of nine outcomes improved: ES .35	Both education and relaxation-based behavioral interventions have been shown to improve important clinical outcomes in adults with asthma	The provision of psychoeducational care is well justified by the existing research	Methodological weaknesses should be rectified in future research (report duration of the intervention; details and severity of the lung disease; random assignment; placebo-type control treatment	Placebo-control is needed to test the relative effect of specific treatments over the generic effect of the psychosocial support from an interested caregiver

Tabulated overview of included reviews

First author	Number of RCT's	Review / Meta	Adherence Interventions	Results / Improvement	Authors' conclusions	Authors' recommendations for practice	Authors' recommendations for research	Authors' other remarks
Devine EC et.al., 1995<br>Hypertension<br>Search 1965-1993<br>88 studies<br>6.581 patients	51 RCT	Meta	psycho-educational care	1) Improved knowledge ES= 1.03<br>2) Medication compliance ES=.74<br>3) Appointment keeping ES=.47	1) Significant large effects were obtained on: knowledge; medication compliance; compliance with appointments<br>2) Effects on weight are unknown (due to methodological weaknesses)	Education, self monitoring of blood pressure and mobilizing psychosocial supports should be considered appropriate nursing interventions for outpatient adults with hypertension	Avoid three current methodological weaknesses: underreporting (sample, duration, e.g.); no multiple blood pressure measures at pretest; few studies compared different types of psychoeducational care	Effects on blood pressure were significantly smaller in studies with multiple pretest measures of blood pressure, (because) blood pressure tends to decrease over time when it is measured on multiple occasions
DiMatteo MR, 2004<br>Various diseases<br>Search 1948-2001<br>122 studies<br>12.010 patients		Meta	Social support	1) Practical social suport: ES=.65<br>2) Emotional social support: ES .30<br>3) Unidimensional social support: ES=.43<br>(other relations with adherence set aside)	1) There is solid quantitative evidence that social support has substantial effects on patient adherence<br>2) Practical social support bears the highest correlation with adherence	1) Practical support is a potentially important target for interventions<br>2) In clinical settings it is important to assess the type and quality of patients' relationships to assist them to receive most benefit from treatment	1) More systematic empirical study is needed, including multidimensional longitudinal research<br>2) Comparisons among various types of acute and chronic conditions to determine exactly how social support works to affect adherence	1) Studies using self report of adherence yield higher correlations (r=.35) than other measures (r=.20)<br>2) At the aggregate level there is no evidence that the mean effect is a result of the use of self-reports
Dodds F et.al., 2000<br>Psychotic disorder<br>Search 1984-1999<br>8 studies<br>543 patients	8 RCT<br>follow-up<br>6 m	Review	Various interventions	1) Improved compliance in 3/8 studies	1) Compliance can be improved by certain, sometimes complex, interventions<br>2) Effective interventions included: individualized behaviour tailoring regimes and compliance therapy	The evidence supports using a combination of daily living activities and medication regimes combined as a behavioural reminder, incorporated as a joint activity and not treated as a separate entity	More larger (field) studies are needed	1) Further efforts are needed in developing effective interventions<br>2) The implicit assumption that knowledge improves compliance is unfounded

Tabulated overview of included reviews

First author	Number of RCT's	Review / Meta	Adherence Interventions	Results / Improvement	Authors' conclusions	Authors' recommendations for practice	Authors' recommendations for research	Authors' other remarks
Dolder ChR et.al., 2003<br>Schizophrenia<br>Search 1980-2001<br>21 studies<br>2394 patients	16 RCT	Review	Educational<br>Behavioral<br>Affective<br>Combinations	1) Improvements in 15 of 23 interventions<br>2) Education effective: ¼ studies<br>3) Behavioral int. effective: 2/2 studies<br>4) Affective int. effective: 4/5 studies<br>5) Combined int. effective: 8/12 studies	1) Education only is least successful<br>2) Combinations were more effective<br>3) Longer interventions are important, and alliance with the therapist	1) Monitor regularly patient's level of adherence<br>2) Detect risk factors for non-adherence<br>3) Implement strategies to address these factors<br>4) Appropriate therapy intensity and duration	1) Effective interventions are needed<br>2) Large samples (for subgroup analyses)<br>3) Use multiple measures of adherence<br>4) More RCT's and long(er) follow-up	1) The active components in multiple strategy interventions are difficult to isolate,<br>2) The use of multiple strategies may dilute what was originally effective in single strategy interventions
Giuffrida A et.al., 1997<br>Various diseases<br>Search 1966-1997<br>11 studies<br>2721 patients	11 RCT	Review	Financial incentives	1) Improved adherence in 10/11 studies<br>2) Financial incentives promoted compliance better than any alternative intervention	1) Financial incentives can significantly reduce non-compliance<br>2) Incentives can be cost-effective, particularly for treatment of infectious disease	In areas of health care where important individual or external effects are associated with non-compliance, monetary incentives may be relatively cost effective (for example: infecting others; development of drug resistance strains)	RCT's outside USA needed (all studies from USA)	It is unknown whether cash or gifts are more effective, but cash payment would be expected to be more effective
Haynes RB et.al., 2005<br>Various diseases<br>Search 1967-2004<br>57 studies<br>10.010 patients	57 RCT<br>6 months follow-up<br>80% follow-up	Review	Various interventions<br>a) for short term treatments<br>b) for long-term treatments	1) Short term treatment: 4/9 interventions effects on adherence and outcome<br>2) Long-term treatment: 26 of 58 interventions effects on adherence, and 18/58 interventions effects on outcomes	1) Improving short term adherence is relatively successful with a variety of simple interventions<br>2) Current methods of improving adherence for chronic health problems are mostly complex and not very effective<br>3) Efforts to improve adherence must be maintained for as long as the treatment is needed: low adherence cannot be 'cured'	Perhaps the most important simple intervention, given its simplicity and effectiveness, is recalling patients who missed appointments, making every effort to keep them in care	1) High priority should be given to fundamental and applied research concerning innovations to assist patients to follow medication prescriptions for long term medical disorders<br>2) Perhaps include patients in the development of new interventions	1) Investigators should join across clinical disciplines to tackle the problem<br>2) Take into account patients' resistance to taking medicines<br>3) The common thread (to complex interventions) is more frequent interaction with patients with attention to adherence

Tabulated overview of included reviews

First author	Number of RCT's	Review / Meta	Adherence Interventions	Results / Improvement	Authors' conclusions	Authors' recommendations for practice	Authors' recommendations for research	Authors' other remarks
Higgins N et.al., 2004<br>Various diseases (older people)<br>Search 1966-2002<br>7 studies<br>1030 patients	6 RCT	Review	1) Medication schedules<br>2) Education<br>3) Combinations	1) Improved adherence: 3/7 studies	1) There is no robust evidence supporting any one type of approach<br>2) Successful interventions are likely to be combinations<br>3) Significant effects tended to have small effects clinically<br>4) Single discrete interventions were disappointing	There is not yet any strong evidence to support the use of any one type of intervention to help improve adherence amongst older people	1) Methodological well-designed RCT's are needed<br>2) Sophisticated combinations of approaches tailored to a patient's individual needs	1) Problem: complex interventions contain multiple elements. It is therefore difficult to conclude what aspects are effective<br>2) None of the interventions tackled the issue that patients might actively choose not to take medication
Iskedjian M et.al., 2002<br>Hypertension<br>Search 1980-1998<br>8 studies<br>11.485 patients		Meta	1) One daily dosing<br>2) Two daily dosing<br>3) Multiple daily doses	1) One dd adherence = 91-93%<br>2) Two dd adherence = 87-91%<br>3) Multiple dd adherence = 83-86%	1) One dd is associated with higher rates of adherence than either two dd's or multiple dd's with antihypertensive medications<br>2) A simple one dd regimen alone may not result in adequate compliance	One way to establish clinical relevance of an intervention is by calculating the number needed to treat (NNT): to have one additional adherent patient, switch 12 patients from MMD to 1DD	None	The medical consequences may be more grave for those patients failing to adhere to 1dd regimens, since missing one dose results in missing the total daily dose
Macharia WM et.al., 1992<br>Various diseases<br>Search 1966-1990<br>23 studies<br>5285 patients	23 RCT	Meta	1) Cuing (reminders)<br>2) Reducing barriers<br>3) Increasing motivation	1) Mailed reminders: OR 2.2 (1.7-2.9)<br>2) Telephone prompts: OR 2.9 (1.9-4.3)<br>3) Orientation statement: OR 2.9 (1.5-5.6)<br>4) Contracting with patients: OR 1.9 (1.0-3.5)<br>5) Prompts from physicians: OR 1.6 (1.4-2.0)	1) In clinic settings broken appointments can be reduced by mail, telephone, or physician reminders, orienting patients to the clinic; or contracting with patients<br>2) Keeping appointments can be an accurate measure of patient compliance	1) The benefit of interventions is greatly influenced by the baseline rate of appointment keeping (NNT)<br>2) Whether an intervention is worthwhile also depends on the consequences of a missed appointment	1) Studies are needed on repeated prompts because a decay in effects may occur<br>2) Effectiveness of reminders for screening in the general population needs further study	The results cannot be safely extrapolated to self-administered treatments; the results concern supervised administration of care

Tabulated overview of included reviews

First author	Number of RCT's	Review / Meta	Adherence Interventions	Results / Improvement	Authors' conclusions	Authors' recommendations for practice	Authors' recommendations for research	Authors' other remarks
Merinder LB, 2000<br>Schizophrenia<br>Search 1966-1997<br>19 studies<br>1718 patients	15 RCT	Review	Patient education	1) Improved knowledge: 6/11 studies<br>2) Improved compliance: 6/11 studies<br>3) Reduced relapse: 3/7 studies	1) Knowledge and compliance can be improved by educational intervention<br>2) No influence of the duration of interventions was found	1) Didactic formats influence knowledge more readily<br>2) Behavioural components are more efficacious in influencing compliance	1) Further methodologically homogeneous and better reported studies are needed<br>2) Better descriptions of interventions<br>3) Comparisons between different interventions (in duration, intensity and educational method)	1) Due to methodological limitations and the results are far from conclusive<br>2) Interventions seem to develop towards the use of a more didactic interactive format (negotiations of individual illness model)
Morrison A et.al., 2000<br>Hypertension<br>Search 1965-1999<br>29 studies<br>12.835 patients	24 RCT	Meta	Various interventions	1) Worksite care: significant effects<br>2) Physician education: sign.effects<br>3) Electronic vial cap: sign.effects<br>4) Patient cards: sign.effects (tentative)<br>5) Calendar packaging: sign.effects (tentative)<br>6) Reminders mailed: insufficient evidence<br>7) Patient education: conflicting results<br>8) Patient counseling: inconclusive<br>9) Self monitoring: ineffective	Results for meeting goal diastolic blood pressure, when evaluated, were generally in accordance with those for adherence	P & T Committees should consider whether compliance programs incorporating effective interventions would benefit their patient populations	Many of the trials lacked blinding (patients and accessors) and this is a potential source of bias	1) In two trials, patient education reduced diastolic blood pressure, but did not improve adherence:<br>2) Perhaps patient education helps lower blood pressure by means independent of drug therapy

Tabulated overview of included reviews

First author	Number of RCT's	Review / Meta	Adherence Interventions	Results / Improvement	Authors' conclusions	Authors' recommendations for practice	Authors' recommendations for research	Authors' other remarks
Mullen PD et.al., 1992<br>Cardiac Care<br>Search 1971-1992<br>28 studies<br>4995 patients		Meta	Patient education	1) Exercise: effects: ES = 0.18<br>2) Diet: effects ES = 0.19<br>3) Smoking: effects not significant<br>4) Drug adherence: effects not significant<br>(blood pressure: effects ES 0.51)<br>(mortality: effects ES = 0.24)	1) Cardiac patient education programs have a measurable impact on blood pressure, mortality, exercise and diet<br>2) Type of communication channel did not influence outcome;<br>3) Applying five principles of education was associated with larger effects.<br>4) No differences were found for number of contacts or total contact hours	The use of educational principles is recommended:<br>reinforce positive behavior;<br>offer feedback (on progress);<br>individualize educational program;<br>facilitate behavior;<br>relevance to the learner's interest and situation	Use of control groups is strongly advised	1) The results suggest that it is not the time per se but how it is spent<br>2) No difference between didactic vs behavioral, apparently because of relatively intensive affective interventions in the didactic group
Newell SA et.al., 1999<br>Cardiovasculair disease<br>Search 1985-1996<br>20 studies<br>4226 patients	20 RCT	Review	1) Patient focused strategies<br>2) Structural strategies<br>3) Physician focused strategies	1) Medication taking: effective 2/6 studies<br>2) Refill compliance: effective: 3/3 studies<br>3) Appointment keeping: effective 8/11 studies	1) Tentative recommendations for many patient focused and structural strategies<br>2) Tentative recommendations against physician focused strategies	The methodological quality of many trials were not optimal, prohibiting strong recommendations	Overcome methodological flaws of current studies:<br>randomized controlled trials<br>follow-up at least 6 months<br>a no-intervention control group<br>adequate sample sizes<br>direct objective measure of compliance or multiple outcome measures	Despite the limitations we believe that this review represents one of the most rigorous that has been conducted of the recent literature

Tabulated overview of included reviews

First author	Number of RCT's	Review / Meta	Adherence Interventions	Results / Improvement	Authors' conclusions	Authors' recommendations for practice	Authors' recommendations for research	Authors' other remarks
Newell SA et.al., 2000<br>Cardiovasculair disease<br>Search 1985-1996<br>18 studies	18 RCT	Review	Various interventions	1) Diet: effective 5/15 studies<br>2) Smoking: effective 1/12 studies<br>3) Exercises: effective 6/9 studies<br>4) Weight-loss: effective 3/7 studies<br>5) Lifestyle: effective ¼ studies<br>6) Screening blood pressure: effective ½<br>7) Stress management: effective 0/1<br>8) Relaxation adherence: effective 4/5	1) Strong recommendations were made for 3 out of 27 interventions: smoking: audiovisual material weight loss: compliance monitoring and feedback for weight loss<br>2) Patient focused strategies showed mixed results<br>3) Tentative recommendations for structural and partner focused strategies<br>4) Physician focused strategies were unanimously unsuccessful	The methodological quality of many trials were not optimal, prohibiting strong recommendations	Overcome methodological flaws of current studies:<br>randomized controlled trials<br>follow-up at least 6 months<br>a no-intervention control group<br>adequate sample sizes<br>direct objective measure of compliance<br>or multiple outcome measures	1) Despite the limitations we believe this review represents one of the most rigorous conducted of the recent literature<br>2) The low quality of studies is disappointing because similar criticisms have been raised before in previous reviews
Nosé M et.al., 2003<br>Schizophrenia<br>Search 1980-2003<br>24 studies<br>3578 patients	14 RCT	Meta	Educational strategies<br>Psychotherapy<br>Prompts<br>Specific services<br>Family interventions	1) Educational strategies: effects OR = 2.41<br>2) Psychotherapy: effectis OR = 2.83<br>3) Prompts: effects OR = 1.87<br>4) Specific services: effects OR = 3.63<br>5) Family interventions: effects OR = 4.45	1) Overall odds ratio is 2.59 for dichotomous outcomes (95% CI 2.21-3.03) and a standardized mean difference of 0,36 for continuous outcomes<br>2) Thus, these interventions more than double the likelihood of adherence to psychotropic medications and to scheduled appointments<br>3) No intervention emerged as predictor of overall treatment effect<br>4) This does not imply that all interventions are equally effective	Community psychiatric services can potentially use effective clinical interventions, backed by scientific evidence, for reducing patient non-adherence	1) Experimental studies have to address the effectiveness of different strategies in large samples.<br>2) Long term follow-up is needed to assess long-term effects<br>3) Trials must adopt high (methodological) standards	Effects of interventions were greater in studies with a short follow-up period (OR 2.27, 95% CI 1.78-2.90) than those with a follow-up of 6 months or more (OR 1.70, 95% CI 1.04-2.78)

Tabulated overview of included reviews

First author	Number of RCT's	Review / Meta	Adherence Interventions	Results / Improvement	Authors' conclusions	Authors' recommendations for practice	Authors' recommendations for research	Authors' other remarks
Pampallona S et.al., 2002<br>Depression<br>Search 1990-1999<br>32 studies<br>12.454 patients	14 RCT	Review	Psychological treatment<br>Patient education<br>Family education<br>Training physicians<br>Training nurses<br>Changing treatment<br>Medication clinics	1) The studies did not give consistent indications of which interventions are effective<br>2) Even looking at contrasts, as we did, does not disentangle the effects of each component	1) By implementing several interventions at the same time, many studies could not provide evidence on the separate effects of the components<br>2) The question is whether all the components are needed in combination<br>3) The trend is that more interventions generally showed a higher adherence rate	Evidence suggests that adherence can be improved	1) Carefully designed clinical trials are needed to clarify the effects of single and combined interventions on adherence.<br>2) Such studies and the interventions should be feasible in busy clinical practice	none
Peterson AM et.al., 2003<br>Hyperlipidemia<br>Search 1966-2000<br>4 studies<br>3077 patients	4 RCT	Meta	Behavioral<br>Educational<br>Combination	1) Behavioral interventions: ES = 0.14<br>2) Educational interventions not single applied<br>3) Combined interventions: ES = 0.03	The interventions had little impact on drug adherence	1) Five (of seven) interventions consisted a change in drug type (from bile acid sequestrants to statins or niacin)<br>2) Given that statins are now regarded as first-line agents, it is difficult to generalize the findings of this study to current treatment regimens	More studies are needed to assess how to improve drug adherence in patients with hyperlipidemia	Traditional interventions to improve drug adherence in patients with hyperlipidemia have little impact

Tabulated overview of included reviews

First author	Number of RCT's	Review / Meta	Adherence Interventions	Results / Improvement	Authors' conclusions	Authors' recommendations for practice	Authors' recommendations for research	Authors' other remarks
Peterson AM et.al., 2003<br>Various diseases<br>Search 1966-2000<br>61 studies<br>18.922 patients	61 RCT	Meta	Behavioral<br>Educational<br>Combination	1) behavioral interventions: ES = 0.07<br>2) educational interventions: ES = 0.11<br>3) combined interventions: ES = 0.08	1) There were no significant differences among behavioral interventions<br>2) There were no significant differences among educational interventions<br>3) Of combined interventions, mail reminders had the largest impact ES=0.38 followed by skill building ES=0.17, packaging ES=0.14 and dosing ES=0.12<br>4) The overall mean increase in medication adherence was 4-11%	For many decades we have searched for that one<br>perfect solution to the problem, however, there does not seem to be any one intervention that robustly enhances adherence, perhaps because so many variables affect a patient's decision to take a drug	1) A standard definition and a standard measure of adherence are needed and a standard format for reporting data<br>2) More studies are needed between adherence and health outcome	1) Roter found an ES between 0.17-0.27,but she included also nonrandomized studies<br>2) Nonrandomized trials may have overestimated the true effects
Richter A et.al., 2003<br>Various diseases<br>Search 1985-2002<br>62 studies		Review	Dose reduction	1) Dose reduction effective in 12/13 studies	1) Reducing the number of daily doses has frequently been shown to provide the patient with better symptom control<br>2) Overall improvements were seen in adherence, quality of life, patient satisfaction and costs	Where reducing the dose frequency is feasible, it may offer benefits for the patient in terms of health outcome and for the health care budget holder in terms of costs (some medications are not suited for reducing dose)	Persistence with medication in chronic diseases remains an area for future research	Probably the single most important action that health care providers can take to improve adherence is to select medications that permit the lowest daily prescribed dose frequency (citation Eisen et.al.1990)

Tabulated overview of included reviews

First author	Number of RCT's	Review / Meta	Adherence Interventions	Results / Improvement	Authors' conclusions	Authors' recommendations for practice	Authors' recommendations for research	Authors' other remarks
Roter DL et.al., 1998<br>Various diseases<br>Search 1977-1994<br>153 studies<br>57.528 patients	116 RCT	Meta	Educational<br>Behavioral<br>Affective<br>Combinations<br>Provider directed	1) Educational interventions ES = 0.23 (dir)*<br>2) Behavioral interventions ES = 0.17 (dir)<br>3) Affective interventions ES = 0.31 (dir)<br>4) Combined interventions ES=0.19-0.34 (dir)<br><br>*) dir = direct measures of adherence	1) Significant effects for all the compliance indicators<br>2) Compliance interventions had a weak to moderate statistical effect<br>3) Smaller effects were evident for improved health outcomes<br>4) No single strategy showed any clear advantage compared with another<br>5) Comprehensive interventions were more effective than single interventions	1) Even small effect sizes can be clinically impressive<br>2) Chronic disease patients, including those with diabetes, hypertension, cancer and mental health problems especially benefited from interventions	1) Compliance intervention studies have been much too narrow and limited<br>2) Compliance interventions should be designed to address the broader spectrum of patient outcomes (including satisfaction, quality of life, empowerment e.g.)	1) Two health education axioms, that people learn in different ways and that a variety of teaching approaches increases learner interest, have been validated<br>2) The effects are of similar magnitude to those generally considered to be successful in medicine
Schedlbauer A et.al., 2004<br>Hyperlipidaemia<br>Search 1972-20003<br>8 studies<br>5943 patients	8 RCT	Review	Simplification drugs<br>patient education/ information<br>intensified care<br>complex behavioral approach	1) Improved adherence: 3/8 studies<br>2) Improved adherence & outcome: 1/8 studies	1) The majority of studies did not increase adherence significantly by informing, reminding and motivating patients<br>2) Particular types of interventions did not seem to be more effective than others	At this stage, no adherence-enhancing intervention can be recommended in clinical practice	1) Measure adherence more detailed and reliable<br>2) RCT's with long-term follow up, combinations of adherence and outcome, rigorous methodology, sufficient power (and economic analyses)<br>3) New interventions with a more patient centered approach	Adherence rates were very variable, adherence rates in the control groups ranged from 23% to 94%

Tabulated overview of included reviews

First author	Number of RCT's	Review / Meta	Adherence Interventions	Results / Improvement	Authors' conclusions	Authors' recommendations for practice	Authors' recommendations for research	Authors' other remarks
Schroeder K et.al., 2004<br>Hypertension<br>Search 1975-2000<br>38 studies<br>15.519 patients	38 RCT	Review	Dosing simplification<br>Patient education<br>Patient motivation<br>Complex interventions	1) Dosing simplification: effects 7/9 studies<br>2) Patient education: mostly unsuccessful<br>3) Patient motivation: effects 10/24<br>4) Complex interventions: effects 8/18 studies	1) Reducing the number of doses appears to be effective, although there is less evidence of an effect on blood pressure reduction<br>2) Some motivational strategies and complex interventions appear promising but we need more evidence on their effect through carefully designed RCT's	Reducing the number of daily doses should be tried as a first line strategy (although there is less evidence of an effect on blood pressure)	1) Larger trials of higher quality are needed that use reliable methods of measuring adherence and investigate the relationship between adherence and blood pressure reduction<br>2) Economic evaluations of interventions are needed	1) For complex interventions it is often difficult to estimate the independent effect of individual interventions<br>2) It remains difficult to disentangle specific adherence effects as opposed to non-specific effects of increased attention
Sharp J et.al., 2005<br>Hemodialysis<br>Search 1970-2003<br>16 studies<br>647 patients	1 RCT	Review	Psychological interventions	Psychological interventions appear to indicate some success	1) Psychological interventions to improve adherence to fluid intake restrictions appear to indicate some success in decreasing IWG = Interdialytic weight gain<br>2) The validity of these findings is circumscribed because of the study designs	If we wish to be more confident that psychological interventions are effective in improving adherence to fluid restrictions in hemodialysis patients, larger well-designed trials are required	1) Larger, well designed, controlled, multicenter trials<br>2) Research should consider applying cognitive behavioral techniques to renal population<br>3) Clearer description of intervention protocols	Combined interventions can enhance treatment effect, however, it is difficult to ascertain which technique is responsible for any detected change

Tabulated overview of included reviews

First author	Number of RCT's	Review / Meta	Adherence Interventions	Results / Improvement	Authors' conclusions	Authors' recommendations for practice	Authors' recommendations for research	Authors' other remarks
Takiya LN et.al., 2004<br>Hypertension<br>Search 1970-2000<br>16 studies<br>2446 patients	16 RCT	Meta	Behavioral<br>Educational<br>Combination	1) Behavioral interventions: effect ES = 0.04<br>2) Educational interventions: heterogeneity<br>3) Ccombined interventions: heterogeneity	1) The ES (0.04) indicates a small non-significant improvement of adherence<br>2) Education and combined: no conclusions due to heterogeneity<br>3) Of behavioral interventions package change was most successful (ES 0.12)<br>4) No single intervention improved adherence over others	1) At this time there is no one particular intervention that provides a significant improvement in adherence rates to antihypertensives<br>2) A patient-specific approach should be modeled	More comparative RCT's are needed to determine the value of a given intervention. 2) Investigate the impact of various patient factors to fully understand the reasons for non-adherence	If patients do not belief in modern medications, do not view hypertension as a significant medical condition and hence do not value the medication their adherence may be affected
Van Dam HA et.al., 2003<br>Diabetes<br>Search 1980-2001<br>8 studies<br>1940 patients	8 RCT	Review	Patient directed interventions<br>Provider directed interventions	Tentative conclusion is that patient directed interventions are more effective than provider directed interventions	The most effective interventions are those with a direct approach to support patients participation in diabetes care and self-care behaviour	Diabetes teams could consider to focus on programmes for directly enhancing patient participation in diabetes care	Well-designed intervention studies are needed on the effects of enhancing patient participation	Changing providers' consulting style into a more patient-centered one proves hard to sustain
Van der Wal MHL et.al., 2005<br>Cardiovascular<br>Search 1988-2003<br>48 studies	8 RCT	Review	Various interventions	Significant improvements of adherence in 12/12 studies	1) The outcomes of the review should be interpreted with caution<br>2) Evidence based interventions to improve compliance in patients with heart failure are scarce and need to be developed and tested	1) Targeting patients at risk<br>2) Recommended are patient related, regimen related and health care provider/ organisation related strategies	1) Interventions that can increase compliance need to be tested in RCT's<br>2) Research is needed to establish the optimal dose of the interventions required	1) From this review it is not clear which part of the interventions was most successful<br>2) Moreover, we do not know what the optimal dose or intensity of the intervention should be

Tabulated overview of included reviews

First author	Number of RCT's	Review / Meta	Adherence Interventions	Results / Improvement	Authors' conclusions	Authors' recommendations for practice	Authors' recommendations for research	Authors' other remarks
Van Eijken M et.al., 2003<br>Various diseases (elderly)<br>Search 1975-2001<br>14 studies<br>4196 patients	14 RCT	Review	Generalised single intervention<br>Generalised combined intervention<br>Tailored combined intervention	1) Generalised single: effects 3/13 interventions<br>2) Generalised combined: effects 1/3 interventions<br>3) Tailored combined: effects 3/7 interventions	1) More than half of the interventions had no effect<br>2) Telephone linked reminder systems achieved the most striking effect<br>3) Tailored combined interventions seemed to have more effects than single and generalised interventions	It might be worthwhile to aim at improving compliance to drug regimens in which non-compliance has the largest clinical consequences	1) Future research should include patient perspectives and shared decision making (concordance)<br>2) Methodological quality of RCT's needs improvement	We were not able to unequivocally define the contents of the interventions, because they were not described in sufficient detail
Vergouwen ACM et.al., 2003<br>Depression<br>Search 1966-2002<br>19 studies<br>5232 patients	19 RCT	Review	Patient education<br>Collaborative care	1) Patient education: effects 2/5 studies<br>2) Collaborative care: effects: 9/11 studies	1) Educational interventions failed to demonstrate a clear benefit<br>2) Collaborative care interventions demonstrated significant improvements in adherence and were associated with clinical benefit	1) We found evidence to support the introduction of interventions to enhance adherence<br>2) Targeting only those patients with persistent symptoms (within 2 months) may be a viable option	Research should attempt to elicit the effects of individual components of collaborative interventions (to reduce avoidable costs)	Probably the improvements (in outcome) resulted from improved quality of care (prescribing) and improved adherence
Vermeire E et.al., 2005<br>Diabetes<br>Search 1966-2002<br>21studies<br>4135 patients	14 RCT	Meta	Various interventions	1) Nurse led interventions: small effects<br>2) Home aids: small effects<br>3) Diabetes education: small effects<br>4) Pharmacist interventions: small effects<br>5) Change dosing/ frequency: small effects	Current effort to improve adherence do not show significant effects nor harm	1) Whether any intervention enhances adherence effectively still remains unanswered<br>2) The majority of authors drew positive conclusions, but it is crucial to notice that significant differences were probably not very clinically relevant	1) The generally accepted methodological rules should be applied<br>2) Adherence should be defined explicitly and measured accordingly<br>3) Compliance research lacks economical evaluations	1) Educational interventions were so poorly described that it was impassible to discern differences or similarities between programmes<br>2) Many interventions were mistakenly called adherence interventions (instead of diabetes care)

Tabulated overview of included reviews

First author	Number of RCT's	Review / Meta	Adherence Interventions	Results / Improvement	Authors' conclusions	Authors' recommendations for practice	Authors' recommendations for research	Authors' other remarks
Yildiz A et.al., 2004<br>Depression<br>Search 1976-2000<br>22 studies<br>1710 patients		Meta	Reduced daily dosing	No significant differences in drop outs were found between 1 or more daily doses	The results suggest that adverse events which are significant enough to result in drop-outs, are not more frequent with 1 daily dosing than multiple daily dosing	A simplified treatment regimen may be practical to increase treatment success rates in depression	Future trials will clarify if there is a particular group of patients for whom once or multiple dosing more beneficial, and if adverse events gives different results than the present report	The rates of drop-outs has limited utility when compared to the rates of adverse events, but numerical data on adverse events were not available
Zygmunt A et.al., 2002<br>Schizophrenia<br>Search 1980-2000<br>39 studies<br>3972 patients	33 RCT	Review	Individual treatment<br>Family therapy<br>Group therapy<br>Community interventions<br>Mixed interventions	1) Individual treatment: effects 2/4 studies<br>2) Family therapy: effects 3/12 studies<br>3) Group therapy: effects 0/2 studies<br>4) Community interventions: effects 3/6 studies<br>5) Mixed interventions: effects 3/9 studies	1) Overall, 13/39 studies (=33%) were effective<br>2) Psychoeducational interventions (only knowledge) were ineffective<br>3) Concrete instructions and problem-solving strategies are useful (behavioral)<br>4) Models of community care are promising<br>5) There was little relation between the duration/intensity and effectiveness.<br>6) No one specific intervention showed overwhelming success over others	1) Interventions should be adapted to the changing realities outside the hospital setting (after discharge)<br>2) Booster sessions are needed because adherence problems are recurring<br>3) We recommend that interventions continue for at least 18 months with quarterly assessment	1) Further theoretical development is needed<br>2) Subtypes of non-adherence (intentional versus unintentional etc.) should be assessed to assign patients to appropriate interventions	1) Multifaceted interventions makes it difficult to identify elements that contributed to success or failure of interventions<br>2) Adherence is typically seen as an individual treatment challenge, rather than one that is amenable to contextual influences and various service strategies. The complexity of these influences also complicates theory development
